# Molecular basis of GDF15 induction and suppression by drugs in cardiomyocytes and cancer cells toward precision medicine

**DOI:** 10.1038/s41598-023-38450-w

**Published:** 2023-07-26

**Authors:** Lisa-Maria Winter, Diana Reinhardt, Ariane Schatter, Vivien Tissen, Heike Wiora, Daniel Gerlach, Ulrike Tontsch-Grunt, Florian Colbatzky, Birgit Stierstorfer, Seong-Wook Yun

**Affiliations:** 1 Boehringer Ingelheim Pharma GmbH & Co KG, Birkendorfer Strasse 65, 88397 Biberach an Der Riß, Germany; 2 Boehringer Ingelheim RCV, GmbH & Co KG, 1120 Vienna, Austria

**Keywords:** Drug safety, Toxicology, Pharmacogenetics

## Abstract

GDF15 has recently emerged as a key driver of the development of various disease conditions including cancer cachexia. Not only the tumor itself but also adverse effects of chemotherapy have been reported to contribute to increased GDF15. Although regulation of GDF15 transcription by BET domain has recently been reported, the molecular mechanisms of GDF15 gene regulation by drugs are still unknown, leaving uncertainty about the safe and effective therapeutic strategies targeting GDF15. We screened various cardiotoxic drugs and BET inhibitors for their effects on GDF15 regulation in human cardiomyocytes and cancer cell lines and analyzed in-house and public gene signature databases. We found that DNA damaging drugs induce GDF15 in cardiomyocytes more strongly than drugs with other modes of action. In cancer cells, GDF15 induction varied depending on drug- and cell type-specific gene signatures including mutations in *PI3KCA, TP53, BRAF* and *MUC16*. GDF15 suppression by BET inhibition is particularly effective in cancer cells with low activity of the PI3K/Akt axis and high extracellular concentrations of pantothenate. Our findings provide insights that the risk for GDF15 overexpression and concomitant cachexia can be reduced by a personalized selection of anticancer drugs and patients for precision medicine.

## Introduction

GDF15 has been found to be a key driver of cachexia syndrome, significant body weight loss that often occurs in chronic diseases including cancer or in case of drug-induced organ injury^1,2^. It is a cytokine that mediates the body response to various stress factors such as oxidative stress, tissue injury and drug toxicity. When binding to GDNF-family receptor α-like (GFRAL), localized mainly in the brain stem, it mediates anorexia, alterations in overall metabolism and energy homeostasis and activation of other endocrine stress responses. In health, these mechanisms alert the body to exo- and endogenous toxins by mitigating the exposure and preventing further organ damage through metabolic reprogramming. In disease such as cancer, GDF15 levels can be elevated up to 100-fold, thereby causing pathological conditions^3,4^.

**Figure 1:**
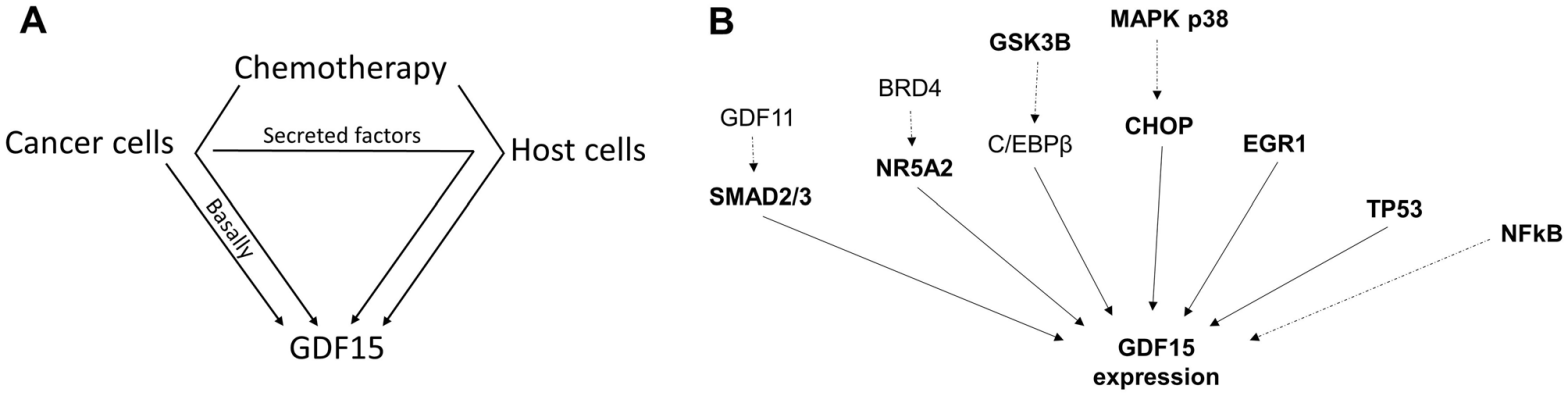
Contributors to increased GDF15 expression in cancer patients. (**A**) Cancer and host cells as sources of increased GDF15 expression. (**B**) Reported molecular pathways upstream of GDF15 induction.

Various types of cancer cells have been reported to heavily secret GDF15 or other factors that induce GDF15 expression in host cells^5,6^. Drug-induced cardiotoxicity is a serious problem often associated with chemotherapy^7^. Anticancer drugs used in chemotherapy can cause cellular stress and death leading to drastic GDF15 induction in both cancer and host cells^8–10^ (Fig. 1A). Cardiomyocytes are one of the host cells known to overexpress GDF15 when exposed to certain cardiotoxic anticancer drugs as well as in heart failure^11–13^. Under these circumstances, elevated GDF15 levels were found in humans as well as in cardiomyocyte cell culture models^14–16^.

Both pro- and anti-tumorigenic pathways were found to directly or indirectly induce GDF15 transcription. EGR1^17^, p53^18^, SMAD2/3^19^, NR5A2^20^, C/EBPβ^21^ and C/EBP homologous protein (CHOP)^22^ were reported to bind directly to the GDF15 promoter and function as transcription activators. GSK3β^23^, NFkB^24^ and p38 MAPK^22^ were identified to indirectly activate GDF15 expression through transcription activators (Fig. 1B).

The latest research suggests that gene expression of GDF15 also depends on bromodomain and extraterminal (BET) proteins, more particularly bromodomain-containing protein 4 (BRD4)^20^. BET proteins have been known to regulate the expression of many genes involved in inflammation, such as cytokines and transcription factors, including the GDF15-activating factors NR5A2 and NFkB^20,25^. By recruiting a variety of coactivators and transcription factors to the transcription site, BET proteins can serve as nucleator for the formation of super-enhancers^26^. In addition, the BET protein BRD4 was reported to increase topoisomerase activity to release accumulated DNA supercoiling ahead of pausing RNA polymerases^27^. These mechanisms might enable the rapid and strong induction of cytokine expression that is needed for a fast reaction and adaptation to the faced stressor. However, in disease states such as cancer or heart failure, BET-dependent stress responses contribute to exaggerated expression of cytokines or genes involved in pathogenesis^28,29^. So far ~30 clinical studies have been conducted with more than 10 BET inhibitors mostly for diverse cancers^30^.

Cachexia has been considered as an epiphenomenal and unpreventable complication with severe cancer progressions and anticancer treatment. Patients who suffer from cachexia are less responsive to anticancer treatment because of their overall weakness^31^. Currently, treatment of cachexia is predominantly focused on non-specific approaches such as to increase appetite or muscle mass^3^. However, the identified causal relations of cachexia with chemotherapy^2,32^, tumor-secreted factors^6^, specific molecular pathways and key molecules^1^ suggest that cachexia is instead a discrete and targetable condition. Specific antibodies targeting GDF15 or its receptor GFRAL have been developed recently to be tested in clinical studies^33^. These approaches could alleviate the direct effects of GDF15 but do not tackle the underlying transcriptional dysregulation. Although GDF15 overexpression has been identified as an etiological key driver of cachexia, little efforts have been made yet to characterize the molecular basis of GDF15 induction and suppression by drugs. A tool to predict whether a patient will react to a particular anticancer drug with GDF15 overexpression could help reduce risks for chemotherapy-driven development of cachexia. Furthermore, the prevention of GDF15 transcription, e.g., by epigenetic intervention, has the potential to effectively counteract cachexia. This study was therefore designed to understand the molecular basis of drug-specific and cell type-specific regulation of GDF15 gene induction and suppression by screening cardiotoxic drugs in human cardiomyocytes and analyzing in-house and public gene signatures database of cancer cell lines.

## Results

### Cardiotoxic drugs induce GDF15 expression in hiPSC-CMs depending on the drug’s mode of action

**Figure 2:**
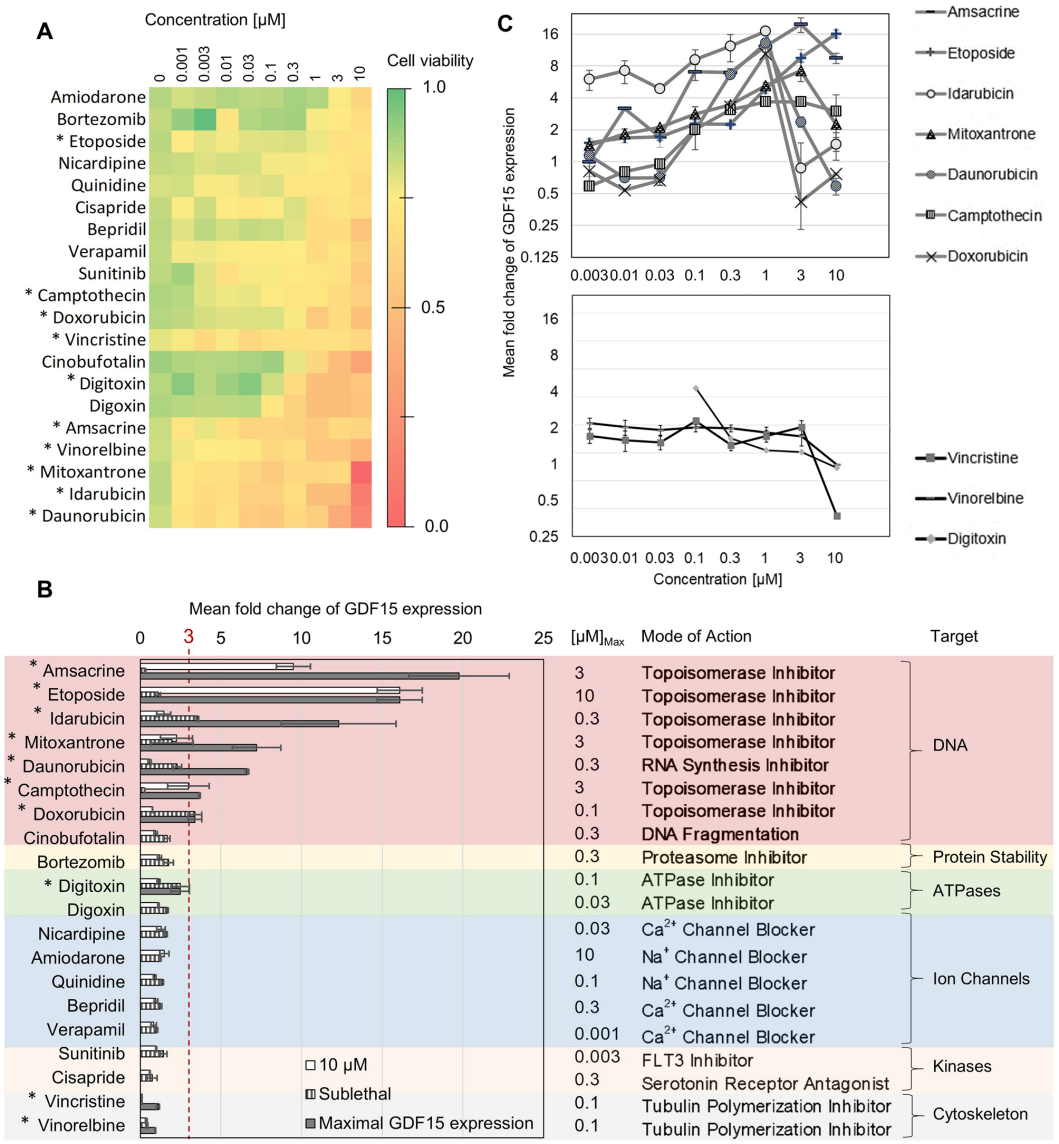
Effect of cardiotoxic drugs on hiPSC-CMs viability and GDF15 secretion and modes of action of applied drugs. (**A**) hiPSC-CMs viability after 48 h treatment with 20 cardiotoxic drugs. (**B**) Fold-change of GDF15 expression upon 48 h treatment with 20 cardiotoxic drugs and their reported modes of action. GDF15 expression upon treatment with 10 µM, sublethal drug concentrations and maximal GDF15 induction (from concentration series depicted in (**C**)). Mean fold-change of 4 biological replicates with a 95% confidence interval were normalized by DMSO-only control. (**C**) Drug-induced GDF15 expression for different drug concentrations. Fold-change of GDF15 protein concentration upon 48 h treatment with ten cardiotoxic drugs (marked with * in **A** and **B**). As the GDF15 concentrations induced by 1 µM idarubicin and 1 µM daunorubicin exceeded the upper limit of quantification, the maximum measurable concentration was used.

As many anticancer drugs are cardiotoxic and GDF15 is a well-known biomarker for heart failure, we employed a human induced pluripotent stem cell-derived cardiomyocytes (hiPSC-CMs) model to investigate the effect of drug-induced cardiotoxicity on GDF15 expression in host tissues. To adequately replicate cardiotoxicity using this model, we selected drugs whose cardiotoxic effect in vivo is based on cytotoxicity to cardiomyocytes. Cell viability screening of 105 known cardiotoxic drugs identified 20 drugs which exerted direct cytotoxicity (Fig. 2A; Supplementary Figure S1). Media concentrations of GDF15 initially measured for the sublethal and highest concentrations (10 µM) of the drugs showed more than threefold increase exclusively by DNA damaging drugs such as anthracyclines (doxorubicin and idarubicin), amsacrine, camptothecin and etoposide (Fig. 2B). An ad-hoc analysis for a broader range of concentrations confirmed the drug-specific GDF15 induction. Within the range of high drug concentrations, reduced GDF15 production caused by general cytotoxicity highly varied (Fig. 2C). In contrast, drugs targeting ion channels, kinases or tubulin polymerization did not induce GDF15 more than 1.6-fold despite their cytotoxic effect on hiPSC-CMs (Fig. 2B,C).

Taken together, we found that induction of GDF15 in hiPSC-CMs by cardio- and cytotoxic drugs varies greatly depending on the drug’s mode of action.

### GDF15 induction by anticancer drugs in cancer cells correlates with the drug’s mode of action and cell type-specific mutation status/expression patterns

To get further insights into how chemotherapy influences GDF15 expression in cancer, we analyzed public gene expression data of NCI-60 cancer cell lines treated with 15 different anticancer drugs^34^. We used linear regression to quantify the cell line’s drug-specific responsiveness regarding GDF15 induction and categorized the cell lines as “responsive” or “resistant”. Comparing the number of responsive cell lines per drug, we found the ratio of responsive cell lines ranged from 4% for sirolimus to 71% for sorafenib (Supplementary Figure S2). Drugs with DNA damaging properties tended to induce GDF15 more strongly than drugs with other modes of action. However, drugs with a similar mode of action did not necessarily lead to similar GDF15 fold-changes (Supplementary Figure S2) and the extent of GDF15 induction by a drug also varied between individual NCI-60 cell lines (Supplementary Figure S3).

**Figure 3:**
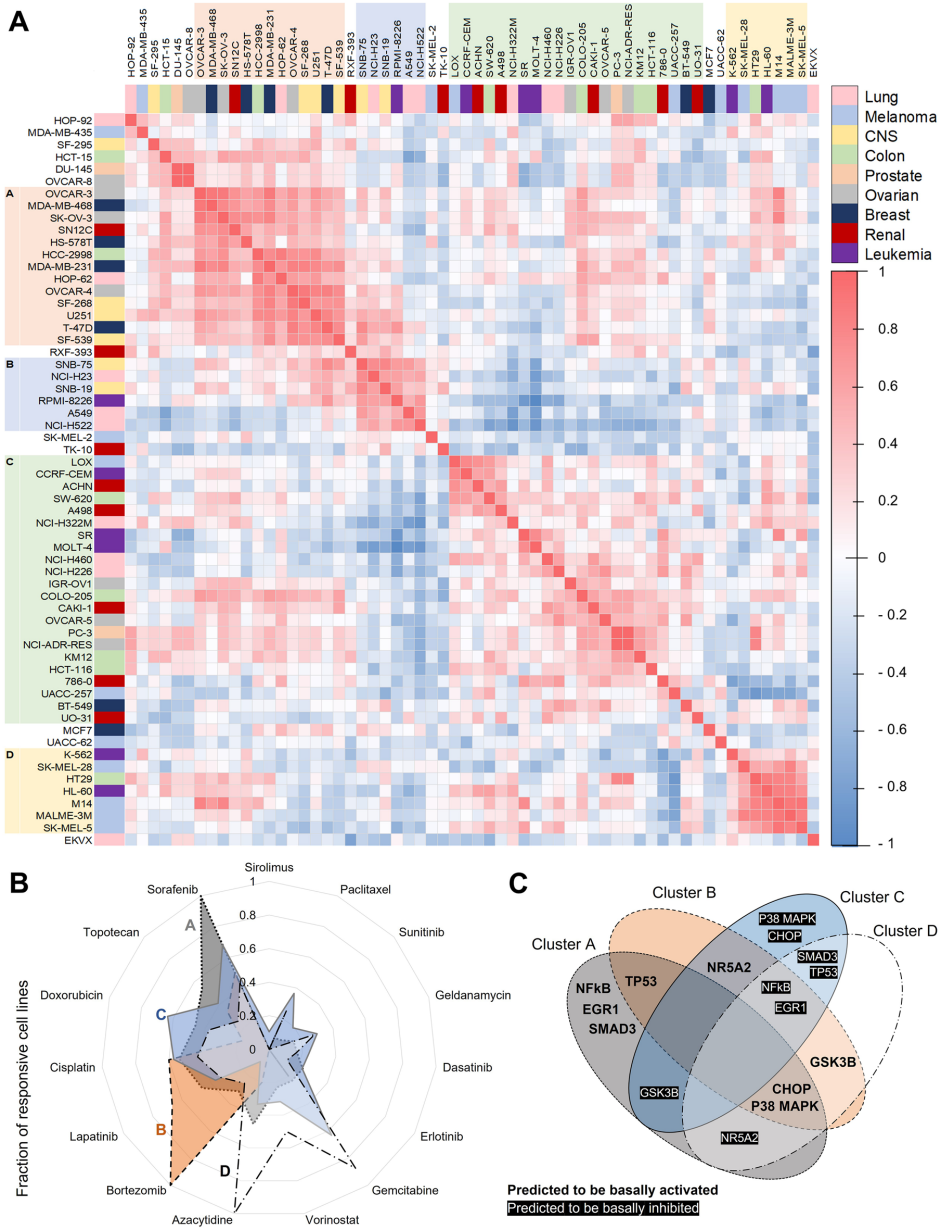
NCI-60 cell line clustering. (**A**) Hierarchically clustered correlation matrix of GDF15 gene expression levels by NCI-60 cell lines upon 15 drug treatments. Pearson correlation coefficients were calculated evaluating linear regression slope of GDF15 expression within the first 24 h of drug treatment. Clustering of cell lines was performed using Weighted Pair Group Method with Arithmetic Mean (WPGMA) algorithm based on Pearson correlation coefficients. Four cell line clusters were identified. Tissue types of cell lines are color coded. (**B**) Drug-specific fraction of responsive NCI-60 cell lines in each identified cell line cluster for 15 anticancer drugs. Responsiveness was assumed when linear regression slope of GDF15 expression within 24 h of drug treatment was > 0.02 and R-squared > 0.5. (**C**) Predicted basally activated or inhibited pathways for the cell line cluster based on identified differences in gene expression.

For a more differentiated view, we generated a Pearson correlation coefficient matrix by calculating pairwise 15-factorial correlation coefficients of the 60 cell lines. By performing a hierarchical clustering on that matrix, we identified four major cell line clusters with every cluster containing cell lines of multiple tissue types (Fig. 3A).

Comparing the relative amount of responsive cell lines per identified cluster, we found that all cell lines of cluster A were responsive to sorafenib, all of cluster B to bortezomib and all of cluster D to azacytidine. Cluster C was most responsive to doxorubicin with 63.6% of its cell lines being responsive (Fig. 3B; Supplementary Figure S4).

Analyzing global genome- and transcriptome data, we tried to find potential explanations for why the four cell line clusters reacted differently to the 15 applied anticancer drugs regarding GDF15 induction. We searched for the most significant differences in expression, mutation and activity of genes that are involved in known GDF15 induction pathways. Additionally, we quantified the correlation of GDF15 and BRD4 fold-change upon treatment. We found that each NCI-60 cell line cluster has distinguishing characteristics that differentiate it from the other clusters (Supplementary Table S1). Cluster C contained only 40.4% cell lines with mutated TP53, whereas 90.9% of the other NCI-60 cell lines showed mutations of that gene. In cluster D, 66.7% of cell lines had mutations in PIK3CB and BRAF genes. Cluster A had the lowest basal GDF15 expression and the lowest correlation of GDF15 and BRD4 fold-change upon treatment. Cluster B had the highest correlation with BRD4 induction and Cluster C the highest basal expression of GDF15.

We used the identified differences in expression of single genes and gene signatures to deduce basal signaling activities in the clusters. Using the QIAGEN Ingenuity Pathway Analysis (IPA), we generated a network comprising key proteins involved in known GDF15 inducing pathways and added reported direct and indirect interactions. We complemented the resulting network with the identified characteristic expression and activity patterns of the four cell line clusters described above. Based on the software-included interactions, we predicted the cluster-specific resulting activities of different GDF15 induction pathways (Fig. 3C).

**Figure 4:**
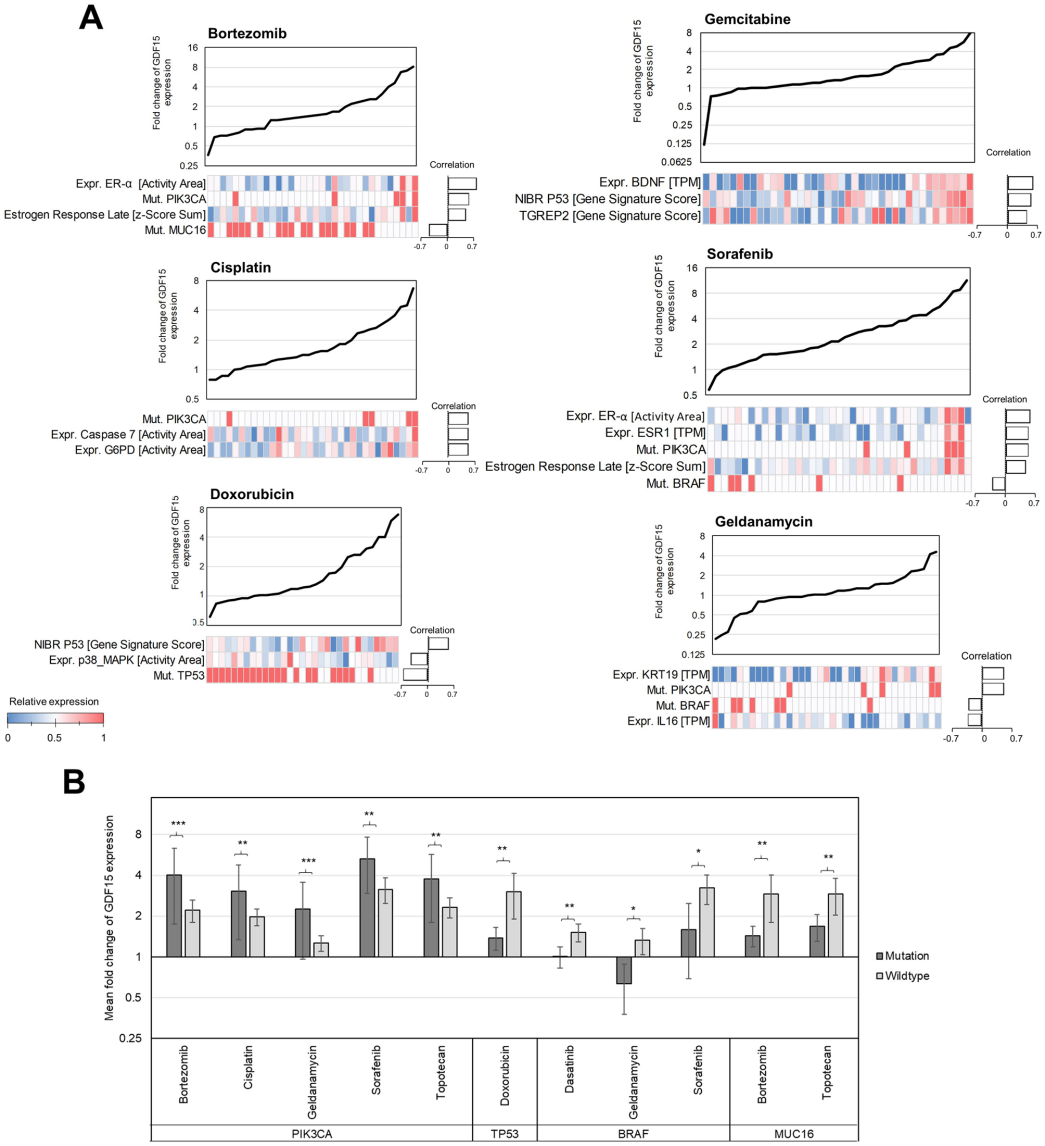
Identified gene signatures that correlate with GDF15 induction upon treatment. (**A**) Drug-specific gene signatures. Cell lines in x and respective correlating molecular factors in y. (**B**) Mutations significantly correlating with GDF15 induction upon treatment. 95% confidence interval, t-test, *p < 0.05, **p < 0.01, ***p < 0.001, ****p < 0.0001.

The identified differences in mutations and gene expressions between cell line clusters indicated that cell-line-specific parameters could correlate with the individual extent of GDF15 induction by certain drugs. By differential analysis and statistical testing of gene expression data from individual cell lines with remarkable GDF15 induction behaviour, we identified additional potentially correlating genes. Together with the previously found cluster-typical expression patterns (Supplementary Table S1), we compiled a list comprised of 79 members whose expression level or mutational status can be expected to correlate with drug-specific GDF15 induction (Supplementary Table S2). Using Pearson correlation coefficients and statistical testing, we squared these cell-line-specific molecular factors with the GDF15 fold-changes for each of the 60 cell lines and all 15 tested drugs. The analysis identified 22 significantly correlating molecular factors for six of the 15 drugs among the 1185 tested possibilities. We found sets of molecular factors for bortezomib, gemcitabine, geldanamycin, cisplatin, doxorubicin and sorafenib that correlated significantly with GDF15 induction in NCI-60 (Fig. 4A). They comprise correlating tendencies in expression of single genes and proteins, gene signatures, hallmark gene sets as well as correlating occurrence of amino acid mutations, which can serve as potential predictors for GDF15 induction upon treatment. Strong GDF15 induction upon bortezomib and sorafenib treatment correlated positively with, inter alia, ER-α protein expression, activity of late estrogen response and mutations in PIK3CA. Cell lines with mutated TP53, low NIBR p53 scores (a gene signature that indicates p53 pathway activity) and high protein expression of p38 MAPK were less prone to GDF15 overexpression upon doxorubicin treatment. Similarly, cell lines with a low NIBR p53 score, BDNF expression and TGREP2 scores (a gene signature that indicates DR5 dependency) induced less GDF15 upon gemcitabine treatment. Mutations in BRAF correlated negatively with the cell line’s tendency for GDF15 induction upon geldanamycin and sorafenib treatment. We also found that cell lines with mutated PIK3CA were more likely to show an increased GDF15 expression across different drug treatments, whereas mutations in TP53, BRAF and MUC16 generally accompanied reduced GDF15 induction (Fig. 4B).

To summarize, our results revealed that anticancer drugs differentially induce GDF15 expression in cancer cell lines depending on the drug and the cell line’s mutational and gene expression profile.

### GDF15 induction upon anticancer drugs is frequently accompanied by BRD4 induction in cancer cells

It has recently been reported that basal GDF15 expression depends on BRD4 in pancreatic and melanoma cancer cell lines^20,35^. However, it remains unknown if transcription of GDF15 relies on BRD4 throughout different cancer cell types. To address that, we searched for correlations of GDF15 and BRD4 linear regression slopes upon treatment in NCI-60 cancer cell lines. When squaring the 15 drug-specific slopes of the 60 cell lines, we found a positive correlation (Pearson correlation coefficient > 0.4) of GDF15 with BRD4 induction in 50.0% and a negative correlation (Pearson correlation coefficient <  − 0.4) in 18.3% of NCI-60 cell lines. Among the different tissue types 75% of renal and 83% of CNS cancer cell lines showed a positive correlation of GDF15 and BRD4 induction. In a comparison of GDF15 and BRD4 induction between all GDF15 inducing drugs, we found a significant positive correlation for lapatinib, erlotinib and sorafenib (all of which are kinase inhibitors) and no significant correlation for other GDF15 inducing drugs.

### BET Inhibition suppresses GDF15 overexpression in a defined subset of cancer cell lines and in hiPSC-CMs

**Figure 5:**
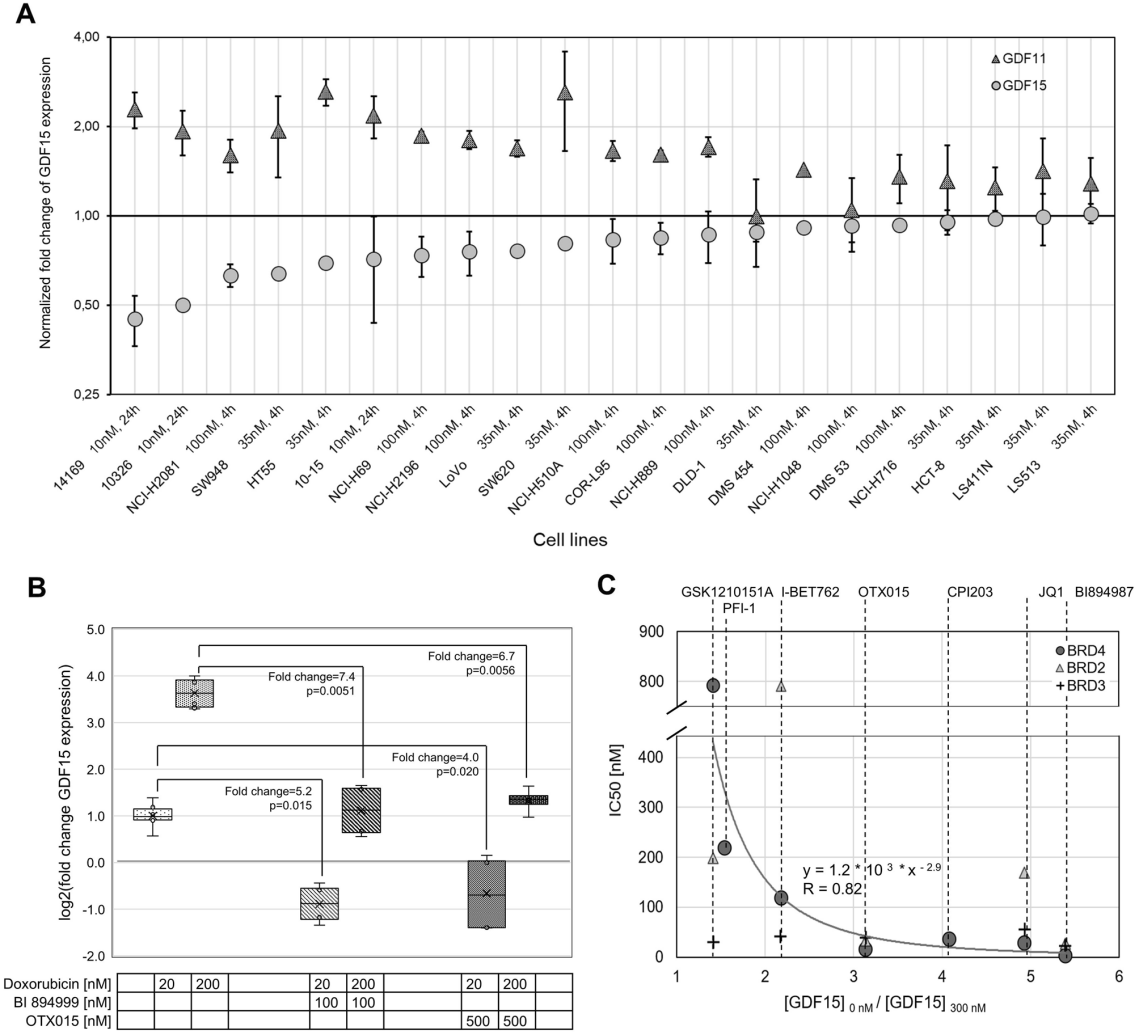
GDF15 gene suppression by BET inhibitors. Effect of BET inhibition on basal GDF15 and GDF11 expression in 21 cancer cell lines and on doxorubicin-induced GDF15 overexpression in hiPSC-CMs. (**A**) Treatment of cancer cell lines with the BET Inhibitor BI 894999 (Supplementary Table S3). Fold-change of relative gene expression. Normalized gene expression analysis performed using RNA sequencing and qPCR, 95% confidence interval. (**B**) Fold-change of GDF15 gene expression in hiPSC-CMs after successive treatment with doxorubicin and BET inhibitors. Normalized to non-treated control. Box plot of quartiles, median, 95% confidence interval, standard one-sided t-test. (**C**) IC50 values for BRD2, BRD3 and BRD4 against fold-change of GDF15 expression in hiPSC-CMs upon treatment with 300 nM of each of seven BET inhibitors. Nonlinear regression.

GDF15 overexpression can be both chemotherapy- and cancer-driven. Various cancer cell types were reported to overexpress GDF15, thereby exacerbating the development of cachexia^5^. Whilst a personalized selection of chemotherapeutic agents has the potential to mitigate chemotherapy-driven GDF15 overexpression in cancer cells, it leaves basal overexpression of GDF15 unaffected. Through the data analysis described above, we found a correlation between BRD4 and GDF15 expression in 50% of NCI-60, indicating that GDF15 expression might depend on BRD4 in those cell lines. To test this further, we analyzed our own gene expression data of 21 lung and colorectal cancer cell lines treated with the BET inhibitor BI 894999^26^. It turned out that in 9 of the 21 treated cell lines, GDF15 expression was significantly reduced through BET inhibition (Fig. 5A). Additionally, we analyzed fold-changes of GDF11 which is well known for its rejuvenating effect but also associated with cachexia, partially through upregulation of GDF15^19^. Interestingly, we found that GDF11 expression was increased by BET inhibition in 19 cell lines with GDF11 and GDF15 fold-changes being inversely correlated (Pearson correlation coefficient − 0.67). The more GDF15 expression was reduced, the more GDF11 expression tended to get increased upon BET inhibition in the respective cell lines (Fig. 5A).

Host cells are known to contribute to elevated GDF15 levels in cachexia patients when exposed to drug toxicity^36^. Many anticancer drugs are cardiotoxic and cardiomyocytes are known to express GDF15 particularly strongly in response to drug toxicity, both in vitro and in vivo^10,11,15^. Hence, it would be important to reduce GDF15 expression not only in cancer cells but also in cardiomyocytes that overexpress GDF15 because of drug toxicity. We tested the BET inhibitors JQ1, I-BET762, PFI-1, I-BET151, CPI203, OTX015, BI 894999 and the structurally closely related BI 894987 in doxorubicin-treated hiPSC-CMs for their influence on drug-induced GDF15 expression. hiPSC-CMs reacted on doxorubicin treatment with up to sevenfold increased GDF15 expression on RNA level. However, when treated with BET inhibitor OTX015 or BI 894999 subsequently, GDF15 overexpression was suppressed (Fig. 5B). To confirm that the suppressed GDF15 expression is not due to general cytotoxicity of the drugs, we measured LDH released into the media to find no increase by the BET inhibitors (Supplementary Figure S5). Similarly, treatment with each of the BET inhibitors JQ1, I-BET762, PFI-1, I-BET151, CPI203, OTX015 and BI 894987 suppressed doxorubicin-induced GDF15 overexpression (Supplementary Figure S6A and S6B; Fig. 5C). We plotted GDF15 fold-changes against the respective reported half maximal inhibitory concentrations (IC_50_) of each BET inhibitor for BRD2, BRD3 and BRD4, whenever available. GDF15 fold-change thereby meant the ratio of GDF15 expression induced by doxorubicin only and doxorubicin combined with BET inhibitor normalized to cell viability. The strongest negative relation was observed between IC_50_(BRD4) and GDF15 fold-change (nonlinear regression, R-squared = 0.82, Fig. 5C). The higher the inhibitory effect against BRD4 was, the less GDF15 induction through doxorubicin treatment was observed.

**Figure 6:**
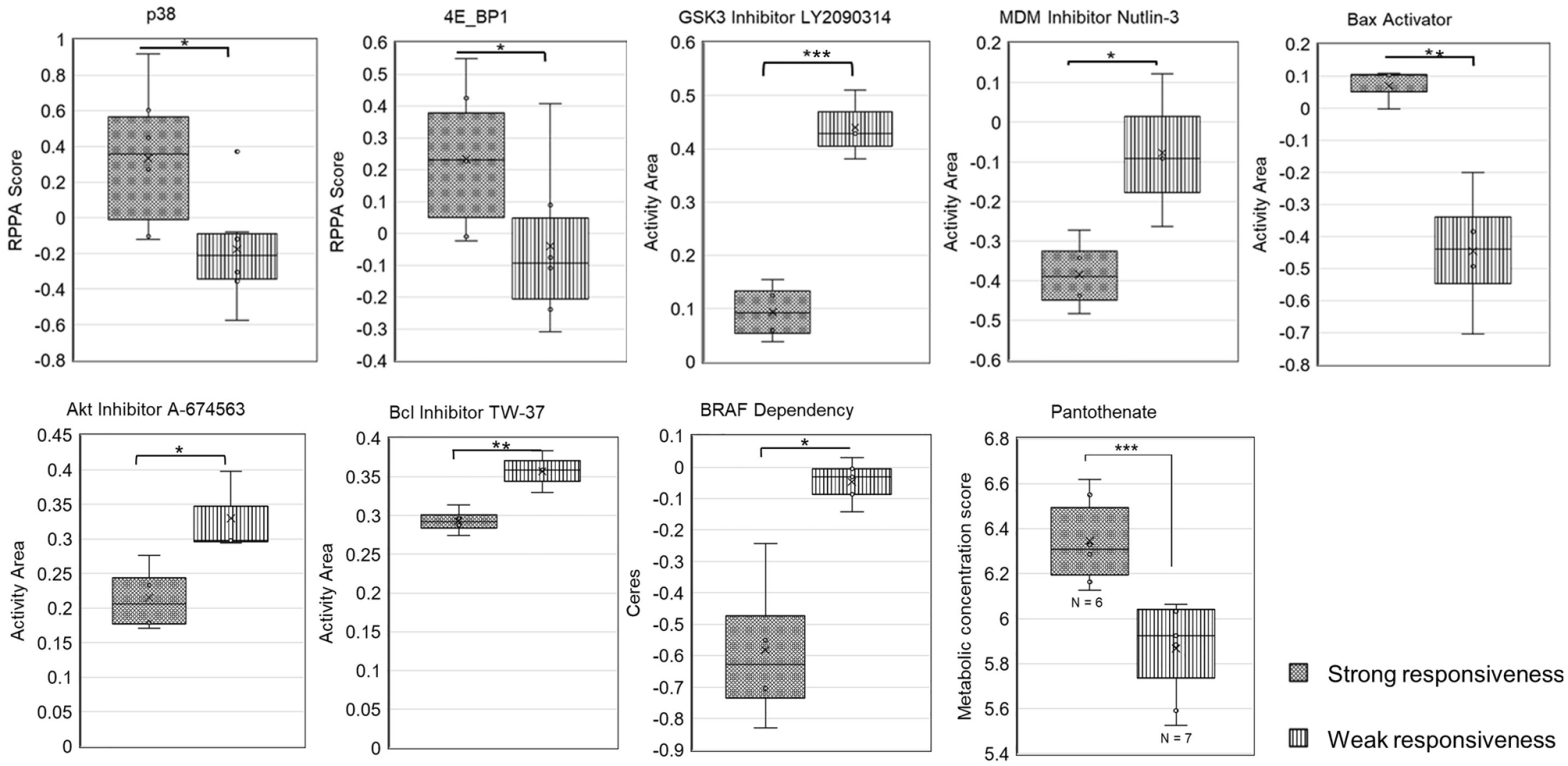
Differential analysis of protein expression patterns, drug sensitivity, gene dependency and pantothenate secretion between strongly and weakly responsive cell lines. Based on GDF15 reduction and GDF11 activation upon BET inhibition. Box plot of quartiles, median, 95% confidence interval, t test, *p < 0.05, **p < 0.01, ***p < 0.001, ****p < 0.0001.

We searched for specific molecular factors that may help predict whether cancer cells reduce GDF15 expression upon BET inhibition and serve as biomarkers for patient selection. Therefore, we categorized the tested lung and colon cancer cell lines in two groups depending on GDF15 reduction and GDF11 induction upon BET inhibition and explored differences in protein expression, gene dependency, drug sensitivity and metabolites (Fig. 6). Cell lines that reduced GDF15 and increased GDF11 expression when treated with BI 894999 had significantly higher p38 and 4E_BP1 protein levels compared to more resistant cell lines. They were less sensitive to GSK3-beta, MDM, Akt and Bcl inhibitors, more sensitive to a Bax activator and more dependent on BRAF. We identified media concentration of pantothenate to correlate with BET inhibitor responsiveness, as its concentration was significantly higher in cell lines that reacted with GDF15 reduction and GDF11 induction on BET inhibition compared to more resistant cell lines (*p* < 0.001, Fig. 6).

To summarize, our results revealed that BET inhibition reduces basal GDF15 expression in a subset of cancer cell lines that share similar biochemical and metabolic properties. Six tested BET inhibitors all suppressed doxorubicin-induced GDF15 expression in hiPSC-CMs to a varying extent depending on their inhibitory effect on BRD4.

## Discussion

Our results show that drug-induced GDF15 overexpression in cardiomyocytes is dependent on the drug’s mechanism. DNA interacting drugs and topoisomerase inhibitors had the strongest inducing effect on GDF15 expression in cardiomyocytes. This is consistent with previously described relations of DNA damaging radiation and transcriptional stress with GDF15 overexpression^16,37,38^. Mitochondrial stress has been known to induce GDF15^39,40^. As DNA damaging drugs often also induce mitochondrial toxicity^41^ and mitochondrial dysfunction appears to be a major cause of anthracycline-induced cardiotoxicity, it is very likely that mitochondrial toxicity is one of the major causes of GDF15 induction we observed in our drug screening in hiPSC-CMs. In NCI-60 cancer cell lines, we observed different effects on GDF15 expression for the two FLT3 inhibitors sorafenib and sunitinib. Whilst sorafenib led to a threefold induction of GDF15 on average, sunitinib did not lead to notable GDF15 regulation in any of the NCI-60 cancer cell lines. A possible explanation for this difference in GDF15 regulation, despite the similar mode of action, is the mitochondrial toxicity that sorafenib was found to induce whereas sunitinib was not^42^. At clinically relevant and even low concentrations, sorafenib increases nitric oxide generation disrupting mitochondrial membrane potential in cancer cells^43,44^.

In the present study, we found that topoisomerase inhibitors, e.g., anthracyclines induce GDF15 expression in hiPSC-CMs strongly. However, BET inhibitors effectively suppressed doxorubicin-induced GDF15 overexpression depending on its inhibitory effect on BRD4. This suggests that the mechanism behind GDF15 overexpression by topoisomerase inhibiting drugs relies on BRD4. We hypothesize that this BRD4-dependence of GDF15 overexpression might be due to BRD4-driven super-enhancer formation. Topoisomerase inhibiting anticancer drugs, such as doxorubicin, impede transcription by increasing DNA supercoiling^45^. Super-enhancer formation would explain how GDF15 overexpression can be achieved, despite the overall transcription inhibition by topoisomerase inhibition. As BRD4 partially promotes transcription by increasing topoisomerase activity^27^, it is likely that BRD4 can compensate for topoisomerase inhibition by an increased recruitment of coactivators and the formation of super-enhancers.

In 50% of NCI-60, we thereby found a correlation between drug-induced GDF15 and BRD4 expression. Reanalysis of our previously published ChIP-seq data from AML cell lines^26^ revealed a reduction of BRD4 bound to putative enhancers in close vicinity to the GDF15 gene locus by the BET inhibitor BI 894999 in a concentration-dependent manner (Supplementary Figure S7). These results suggest that the BRD4-dependence observed for doxorubicin-induced GDF15 overexpression in cardiomyocytes also applies to those cancer cell lines.

Besides drug-induced GDF15 overexpression, some tumors contribute to elevated GDF15 levels in the patients by heavy GDF15 secretion, independently of anticancer treatment^5^. We showed that this basal GDF15 expression gets significantly reduced upon BET inhibition using BI 894999 in a subset of tested cancer cell lines, in which concomitant upregulation of GDF11 was observed. To confirm whether this negative correlation between GDF15 and GDF11 is the result of target-specific inhibition of BRD4 or due to a non-specific effect of the drug on other targets, we looked up literature for gene expression profiling after BRD4 gene knockdown in cancer cell lines. Wu et al.’s study included pan-BRD4 knockdown in MDA-MB-231 and MCF-7 cell lines using siRNA^46^. The retrieved data for GDF15 and GDF11 genes showed a negative correlation between the 2 genes in MDA-MB-231 cells, whereas both genes’ expression was not affected in MCF-7 despite similar reductions of BRD4 expression in both cell lines. This finding is in accord with our findings confirming the up-regulation of GDF11 can be caused by BET inhibition in certain types of cancers and negatively correlated with GDF15. Although BET inhibition also induced GDF11 expression which was described to promote weight loss partially through GDF15^19^, the BET inhibitor JQ1 was found to counteract cancer-driven cachexia in mice suggesting that the advantageous effects of BET inhibition for the alleviation of cachexia clearly predominate^47^. In that mouse study, however, neither GDF15 nor GDF11 level was analyzed. The interplay of GDF11 and GDF15 in cachexia and the potential effects of GDF11 induction by BET inhibition in that context still remain to be further elucidated. By differential analysis, we found that the subset of tested cancer cell lines that responded to BET inhibition with GDF15 reduction and GDF11 induction show a higher activity of p38 MAPK and a significantly lower activity of and dependency on proteins involved in the PI3K/Akt pathway, namely GSK3B, Akt, Bcl and B-Raf, compared to the weakly responding cell lines. It is in accordance with the finding that PI3K pathway activation promotes resistance of neuroblastoma to BET inhibition which was discovered by Iniguez et al. through genome-scale screening and multiomics analysis^48^. NF-kB and p53, two of the seven reported GDF15-inducing pathways, are directly or indirectly activated by GSK3B and Akt^49,50^. Based of the observed low sensitivity to GSK3B and Akt inhibition, we presume that the BET inhibitor responsive cell lines have low basal activities of GSK3B and Akt so that NF-kB and p53 can be expected to have low activities in this subset of cell lines, too. Their high sensitivity to Bax activation might be due to this presumed low activity of p53, as Bax is activated by p53 ^51^. We searched for metabolic markers that reflect the cell line’s pathway activities related to the susceptibility of its GDF15 expression to BET inhibition. We found high basal concentrations of pantothenate to correlate significantly with effective GDF15 reduction upon BET inhibition. Pantothenate kinase 1 involved in CoA synthesis from its precursor pantothenate has been known to be transcriptionally activated by p53, and PI3K signaling stimulates CoA synthesis^52,53^. Hence, high pantothenate concentration might be due to low activity of p53 and PI3K signaling, which is consistent with the results of our differential gene expression analysis discussed above. Variable efficacy and dose-limiting toxic effects of BET inhibitors such as thrombocytopenia are commonly observed in preclinical and clinical studies and strongly imply that the discovery of biomarkers that can help predict therapeutic response is a prerequisite for effective and safe treatment using BET inhibitors^54^. The observed differences in extracellular pantothenate concentration indicate that pantothenate could be developed into a potential biomarker for the selection of patients who would benefit from GDF15 reduction upon BET inhibition.

We found that NCI-60 cancer cell lines can be classified in clusters according to the extent of GDF15 induction by different drugs. We were able to attribute the differences in GDF15 induction to activity patterns of GDF15-inducing pathway. As most GDF15-inducing pathways are involved in cancer genesis, they are often directly targeted by anticancer drugs^55^. Due to the heterogeneity of dysregulations in cancer, it can be assumed that every cancer cell line comes with a distinct setting of GDF15-inducing pathway activities. It is therefore expected that a drug leads to varying extents of GDF15 induction depending on the cell line’s basal pathway activities and how they interplay with the drug’s mode of action. This also points out the importance of establishing precision medicine tailored to the individual requirements. We identified six drug-specific gene signatures, consisting of specific genes whose expressional or mutational status significantly correlate with the cell line’s tendency to overexpress GDF15 upon drug treatment (Fig. 4A). Those gene signatures could be used to predict the effect of a drug on the individual regulation of GDF15 expression. Our data were, however, obtained from in vitro cell culture studies. Biological response of cells in vivo could be different. Also, the 60 cancer cell lines we included in our correlation analysis might not sufficiently represent the variety of cancers. With further data analysis using larger sample sizes and additional validation strategies employing patient samples, the identified gene signatures could be refined and used for a personalized selection of chemotherapy agents and patients, depending on pheno- and genotype of the tumor. Thus, chemotherapy-induced GDF15 overexpression followed by the concomitant risk for developing cachexia could be reduced.

## Materials and methods

### Cell culture

hiPSC-CMs were prepared according to the method described in ref.^56^ with minor modifications, using hiPSC lines SB-AD02-Cl02 and SFC086-03–01 obtained from the StemBANCC consortium. Briefly, after differentiation by CHIR99021 (Biomol, Cay13122-5) and Wnt-C59 (Selleck Chemicals, S7037) in CDM-3 (chemically defined RPMI1640 medium containing albumin and ascorbic acid) and metabolic selection with lactate, hiPSC-CMs were plated at 3 × 10^4^ cells/well on 96-well plates or 1 × 10^6^ cells/well on 6-well plates and cultured in CDM3-M medium (RPMI1640 medium, no glucose (Gibco, 11879020) supplemented with 500 µg/ml recombinant human albumin (Sigma Aldrich, A9731), 4 mM sodium L-lactate (Sigma Aldrich, A71718), 1 mM sodium pyruvate (Sigma Aldrich P5280), 213 µg/ml L-ascorbic acid 2-phosphate (Sigma Aldrich, A8960), 10 mM D-galactose (Carl Roth, 4987.2), 200 ng/ml triiodo-L-thyronine (Sigma Aldrich, T6397), 20 µg/ml Insulin (Invitrogen, RP-10908) and 1 × chemically defined lipid concentrate (Gibco, 11905031)) containing 20% FCS (Lonza, DE14-701F) for 24 h before switching to CDM3-M without FCS.

Cancer cell lines were authenticated by short tandem repeat analysis performed according to the manufacturer’s instructions and treated with 10 nM, 35 nM or 100 nM BI 894999 BET inhibitor for 4 h or 24 h (Supplementary Table S3). RNA^57^ and DNA sequencing^58^ was performed as described previously.

### Drug treatment

Cardiotoxic drug (SCREEN-WELL® Cardiotoxicity library, BML-2850, ENZO) library was prepared by serial dilution of 10 mM stocks in DMSO to 9 different concentrations ranging from 0.001 to 10 mM, which were further diluted into assay plates to final concentrations ranging from 0.001 to 10 µM. BI894987 (Boehringer Ingelheim), BI 894999 (Boehringer Ingelheim), CPI203 (Sigma-Aldrich, SML1212), GSK1210151A (Sigma-Aldrich, SML0666), I-BET762 (Cayman Chemical, Cay10676), JQ1 (Cayman Chemical, Cay11187), OTX015 (eNovation Chemicals LLC, D372528), PFI-1 (Adooq Bioscience, A12545) and Doxorubicin (Sigma-Aldrich, D1515) were dissolved in DMSO to make 30 mM or 10 mM stocks which were serially diluted in DMSO and assay media to concentrations as indicated. The final concentration of DMSO for hiPSC-CMs assay was 0.1%. To investigate the influence of BET inhibitor treatment on doxorubicin induced GDF15 mRNA expression, hiPSC-CMs were pre-treated with 20 nM or 200 nM doxorubicin for 48 h and kept in medium without compound for 24 h before 24 h treatment with either 100 nM BI 894999 or 500 nM OTX015. We screened the BET inhibitors JQ1, I-BET762, PFI-1, I-BET151, CPI203, OTX015 and BI 894987 for their effect on doxorubicin-induced GDF15 secretion in hiPSC-CMs by simultaneous treatment with 100 nM doxorubicin and 0.1 to 1000 nM of each BET inhibitor. Cancer cell lines were treated with 10 nM, 35 nM or 100 nM of BI 894999 for 4 h or 24 h (Supplementary Table S3).

### Cell viability assay

After drug treatment, cell culture medium was replaced with fresh medium containing 0.014 mg/ml resazurin (Sigma, R7017) and incubated for 2 h at 37 °C. The fluorescence of resorufin generated by the cells was measured on a BioTek Synergy HTX microplate reader equipped with an excitation filter 530/25 nm and an emission filter 590/35 nm. After correction for background medium fluorescence and averaging of biological replicates, we calculated cell viability relative to untreated control for each compound concentration.

### GDF15 expression analysis

For GDF15 mRNA quantification, total RNA was isolated from the cells using an RNeasy kit (Qiagen, 74106) to synthesize cDNA using a Taqman Reverse Transcription kit (Applied Biosystems, N8080234). Quantitative real-time PCR analysis was performed using QuantiNova SYBR Green PCR Kit (Qiagen, 208052) on a CFX96 Real-Time System (Bio-Rad) under the following amplification conditions: 95 °C for 2 min followed by 40 cycles of 95 °C for 5 s and 60 °C for 10 s. The primer sequences are GDF15-F: 5′-GAGCTGGGAAGATTCGAACA-3′, GDF15-R: 5′-AGAGATACGCAGGTGCAGGT-3′, ACTB-F: 5′-GTCTTCCCCTCCATCGTG-3′ and ACTB-R: 5′-AGGGTGAGGATGCCTCTCTT-3′. PCR data were analyzed using a modified relative expression software tool (REST) and the expression level of GDF15 gene was normalized to that of β-actin. For secreted GDF15 protein quantification, cell culture medium was collected after drug treatment and stored at − 20 °C until ELISA was performed using Human GDF-15 ELISA Kit (Abcam, ab155432) according to the manufacturer’s instruction. Resulting GDF15 concentrations were normalized to non-treated control and respective relative cell viability.

### Data collection of cancer cell line properties

We obtained basal gene expression values and mutational status of all cancer cell lines from our in-house cancer cell line database. Gene expression data of NCI-60 after compound treatment, basal protein expression, drug sensitivity data, gene dependency data, metabolite expression and compound specifications such as IC50 values and modes of action were collected from different studies and data bases, as described in the supplementary information.

### Clustering

Each NCI-60 cell line was assigned to an array containing 15 drug-specific linear regression slopes of GDF15 mean fold-change. We calculated the Pearson correlation coefficient for every cell line pair to build a 60 × 60 correlation matrix. For agglomerative hierarchical clustering, we applied a “Weighted Pair Group Method with Arithmetic Mean” (WPGMA) algorithm to the resulting correlation matrix. The identified clusters represent groups of cell lines that are closely positioned to each other in this 15-dimensional data set and therefore have more similarities regarding GDF15 expression upon treatment with the 15 drugs compared to cell lines of other clusters.

### Differential analysis

The bioinformatics tool CLIFF^59^ was used for differential analysis. We searched for significant differences between the four identified NCI-60 cell line clusters in terms of protein expression, gene signature and hallmark gene set scores and mutations. Besides in-house gene expression and DNA sequencing data, we extracted protein expression values from a public data set^60^. We applied a modified t-test to identify proteins that differed significantly in their expression level between the different cell line clusters. We included proteins with p-values from 0.001 to 0.05 among which are involved in pathways upstream of GDF15 regulation or reported targets of any of the tested drugs. All the proteins with p-values below 0.001 were included regardless of their involvement in GDF15 regulation or drug mechanisms. Differences in mutation prevalence were quantified using Fisher’s exact test and included whenever the p-value was below 0.05 and the respective gene is involved in GDF15 regulation or is a known target of a tested drug. We additionally deduced specific protein and pathway activities from gene and protein expression data using reported gene signatures and hallmark gene set scores. Detailed references are described in the supplementary information. The resulting scores were additionally used for comparison of the cell line clusters, whenever p-values were below 0.05. GDF15 gene expression values were extracted from NCI-60 Transcriptional Pharmacodynamics Workbench^34^. We compared basal GDF15 gene expression and correlation coefficients of GDF15 and BRD4 induction upon treatment between cell lines of different clusters using standard two-sided t-test. Differences in metabolite concentration between cancer cell lines with strong and weak responsiveness to BET inhibition regarding GDF15 and GDF11 (Supplementary Table S4) were assessed using modified t-tests and considered as significant whenever the p-value was below 0.05 and the individual data points of the two groups did not overlap.

### Correlation analysis

To identify potential candidates for correlating molecular factors, we first performed differential analysis with three NCI-60 cell lines that showed exceptionally high or low GDF15 induction upon treatment, NCI-H460, UACC-257 and MCF7. Genes and proteins that stood out in these cell lines in terms of expression or mutations were included for further analysis as well as previously identified distinguishing genes, proteins and scores of the NCI-60 cell line clusters. Pearson correlation coefficients were used to compare gene, protein, gene signature and hallmark gene set expressions with drug-specific GDF15 fold-change and slope upon treatment. We selected those molecular factors whose Pearson correlation coefficients of both GDF15 fold-change and slope were greater than 0.4 (Supplementary Table S1). Additionally, we sorted out all factors whose correlation with GDF15 fold-change was based only on individual outliers instead of a continuous tendency. To identify correlating mutations, we performed a standard two-sided t-test and considered differences with p-values below 0.05 for both GDF15 fold-change and slope as significant.

### Activity prediction analysis

Using Ingenuity Pathway Analysis (QIAGEN), we generated a network consisting of 44 proteins involved in pathways upstream of GDF15. Depending on the studied cell line cluster, this network was supplemented with respective characteristic expression patterns. We connected the entered proteins (Supplementary Table S5) with direct and indirect interactions that are recorded in the QIAGEN Knowledge Base. To the resulting network, we added the characteristic gene expression patterns and activities that we obtained from differential analysis and used the Molecule Activity Predictor to deduce basal signaling states.

## Supplementary Information

Supplementary Information.

## Data Availability

RNA sequencing data used in this study are available in Gene Expression Omnibus (GEO) database under the accession numbers GSE210542 and GSE183214.
