# Supplementary material for: Molecular basis of GDF15 induction and suppression by drugs in cardiomyocytes and cancer cells toward precision medicine
Source: Sci Rep. 2023 Jul 26;13:12061. doi: 10.1038/s41598-023-38450-w (PMC10372009; doi:10.1038/s41598-023-38450-w)
Supplement: Supplementary file 1 — Supplementary Information. [file 41598_2023_38450_MOESM1_ESM.pdf]

SUPPLEMENTARY INFORMATION

Title

Molecular basis of GDF15 induction and suppression by drugs in cardiomyocytes and cancer cells toward precision medicine

Authors

Lisa-Maria Winter[1], Ariane Schatter[1], Diana Reinhardt[1], Vivien Tissen[1], Heike Wiora[1], Daniel Gerlach[2], Ulrike Tontsch-Grunt[2], Florian Colbatzky[1], Birgit Stierstorfer[1] and Seong-Wook Yun[1]*

[1] Boehringer Ingelheim Pharma GmbH & Co KG, 88397 Biberach an der Riß, Germany

[2] Boehringer Ingelheim RCV GmbH & Co KG, 1120 Vienna, Austria

* Correspondence to: Seong-Wook Yun, Nonclinical Drug Safety, Boehringer Ingelheim Pharma GmbH & Co KG, Birkendorfer Strasse 65, 88397 Biberach an der Riß, Germany. Tel: +49 7351 54 145634. Email: seong-wook.yun@boehringer-ingelheim.com

Supplementary Figures

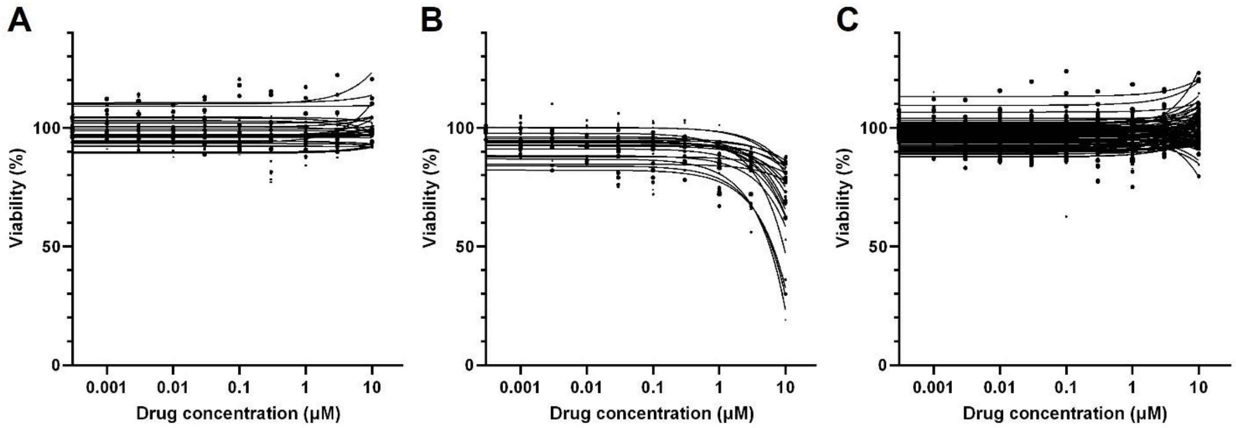

Supplementary Figure S1. Effect of cardiotoxic drugs on hiPSC-CMs viability. **(A)** Non-cardiotoxic control drugs (n=25). **(B)** The identified 20 drugs which showed cytotoxicity within the concentration range between 0.001 and 10 µM). **(C)** The rest of cardiotoxic drugs (n=85). Simple linear regression was applied to the data using GraphPad Prism.

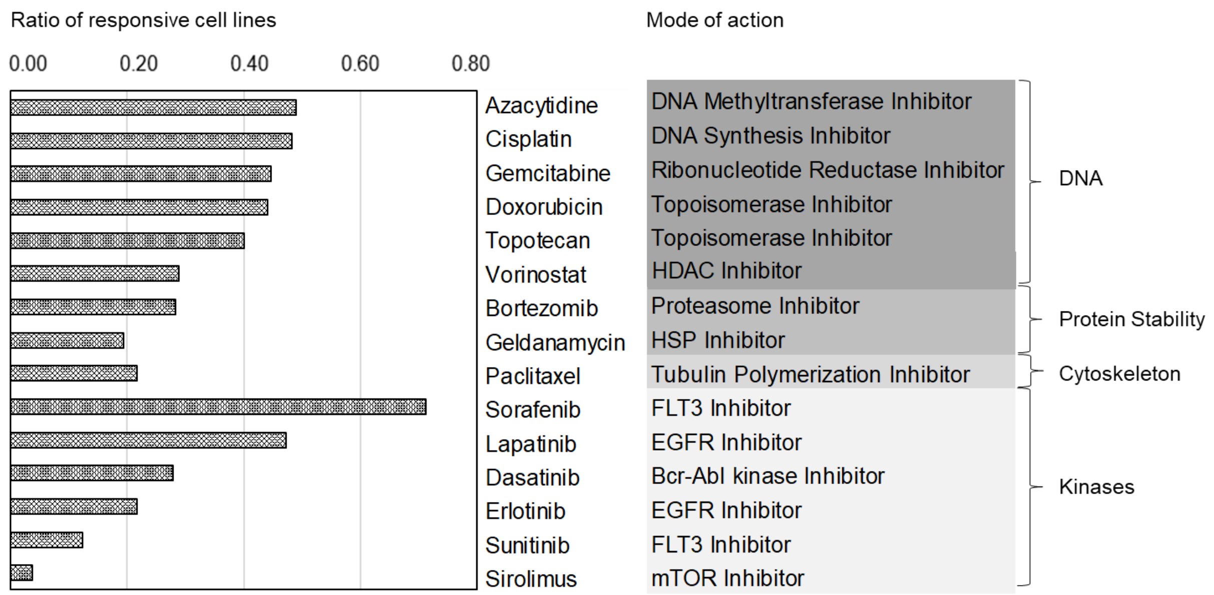

Supplementary Figure S2. Effect of 15 anticancer drugs on GDF15 expression by NCI-60 upon treatment. Ratio of responsive cell lines for 15 drugs. Responsiveness was assumed when linear regression slope of GDF15 expression within the first 24 h of drug treatment was > 0.02 and R-squared > 0.5, Mode of action of applied anticancer drugs [1].

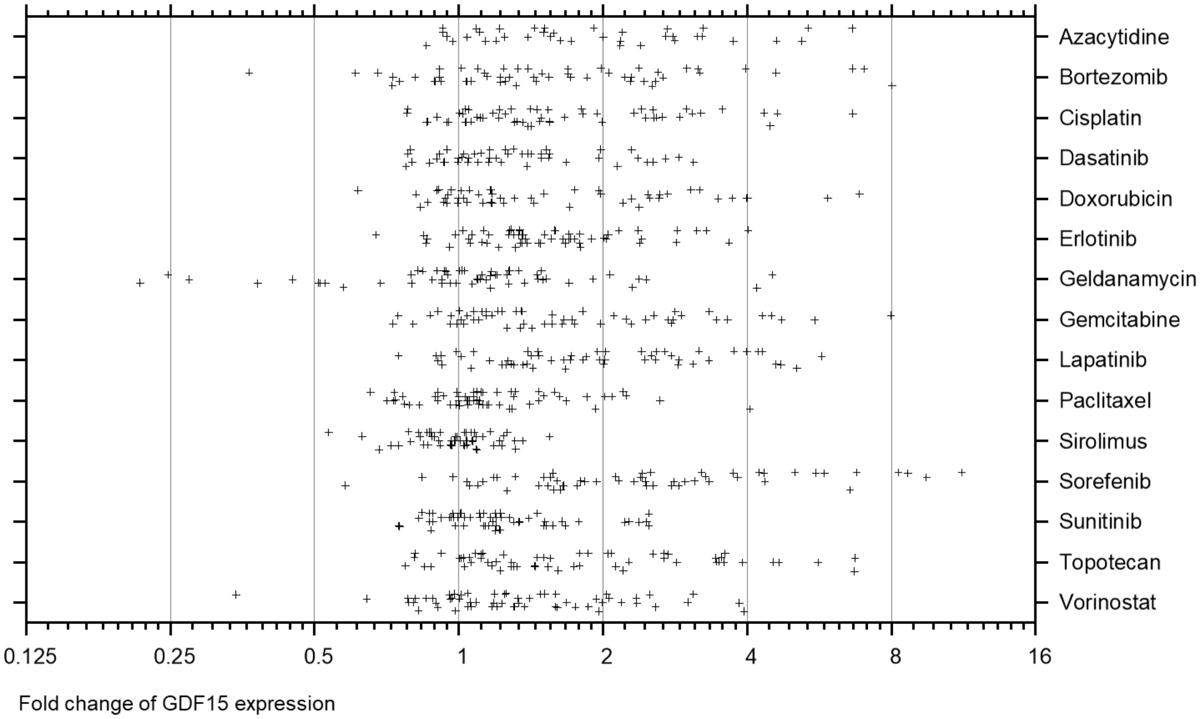

Supplementary Figure S3. GDF15 fold-change after 24 h drug treatment of NCI-60.

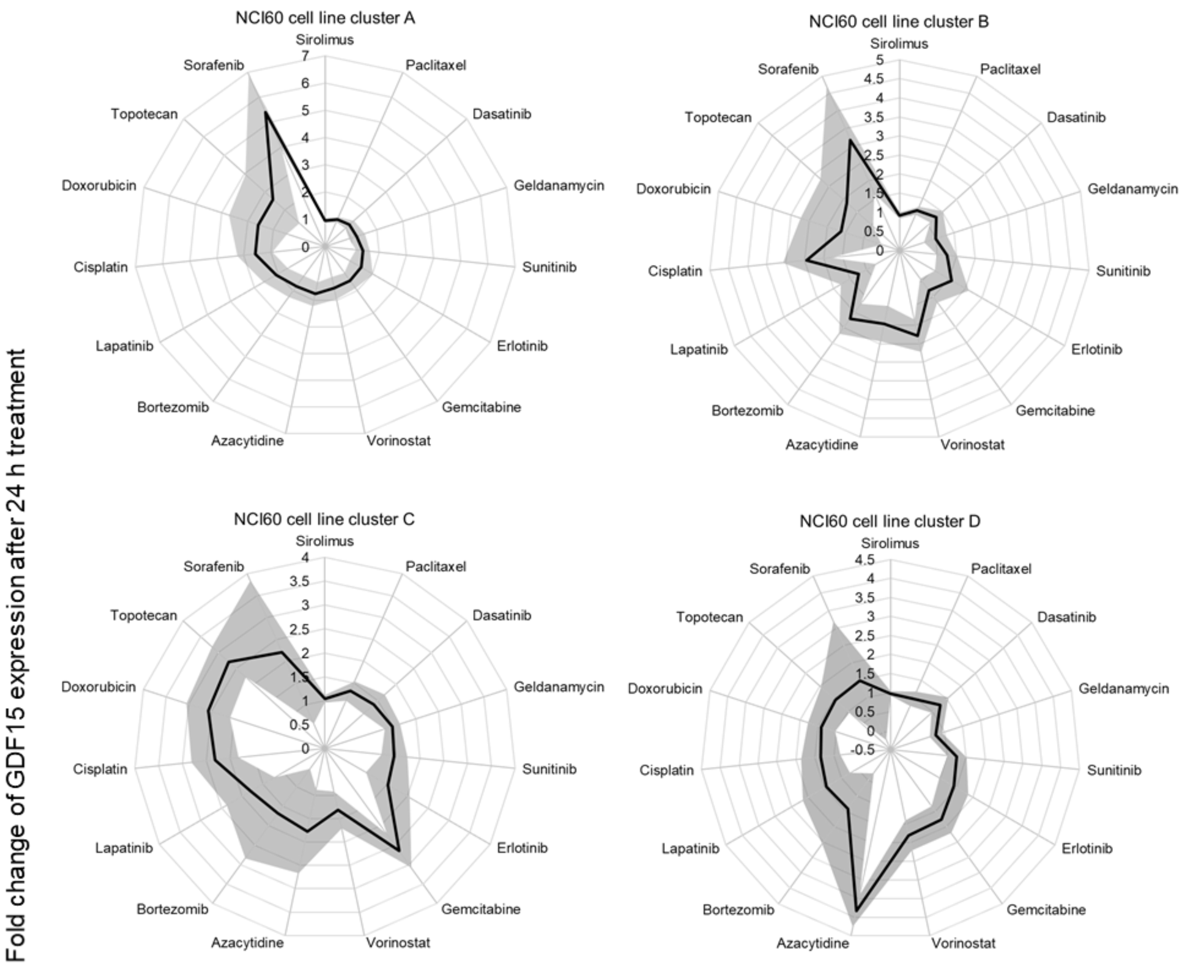

Supplementary Figure S4. GDF15 fold-change in identified NCI-60 cell line clusters upon treatment with 15 anticancer drugs. 95% confidence interval.

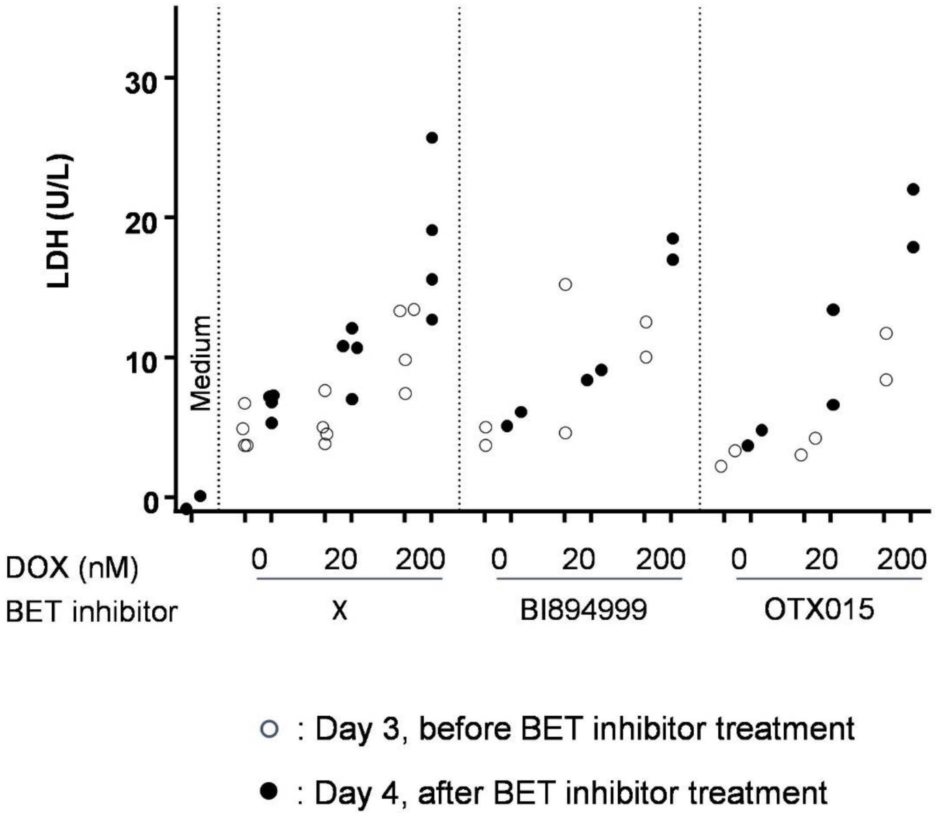

Supplementary Figure S5. LDH release from hiPSC-CM. LDH released into the media was measured on a Cobas 6000 c501 autoanalyzer (Roche Diagnostics).

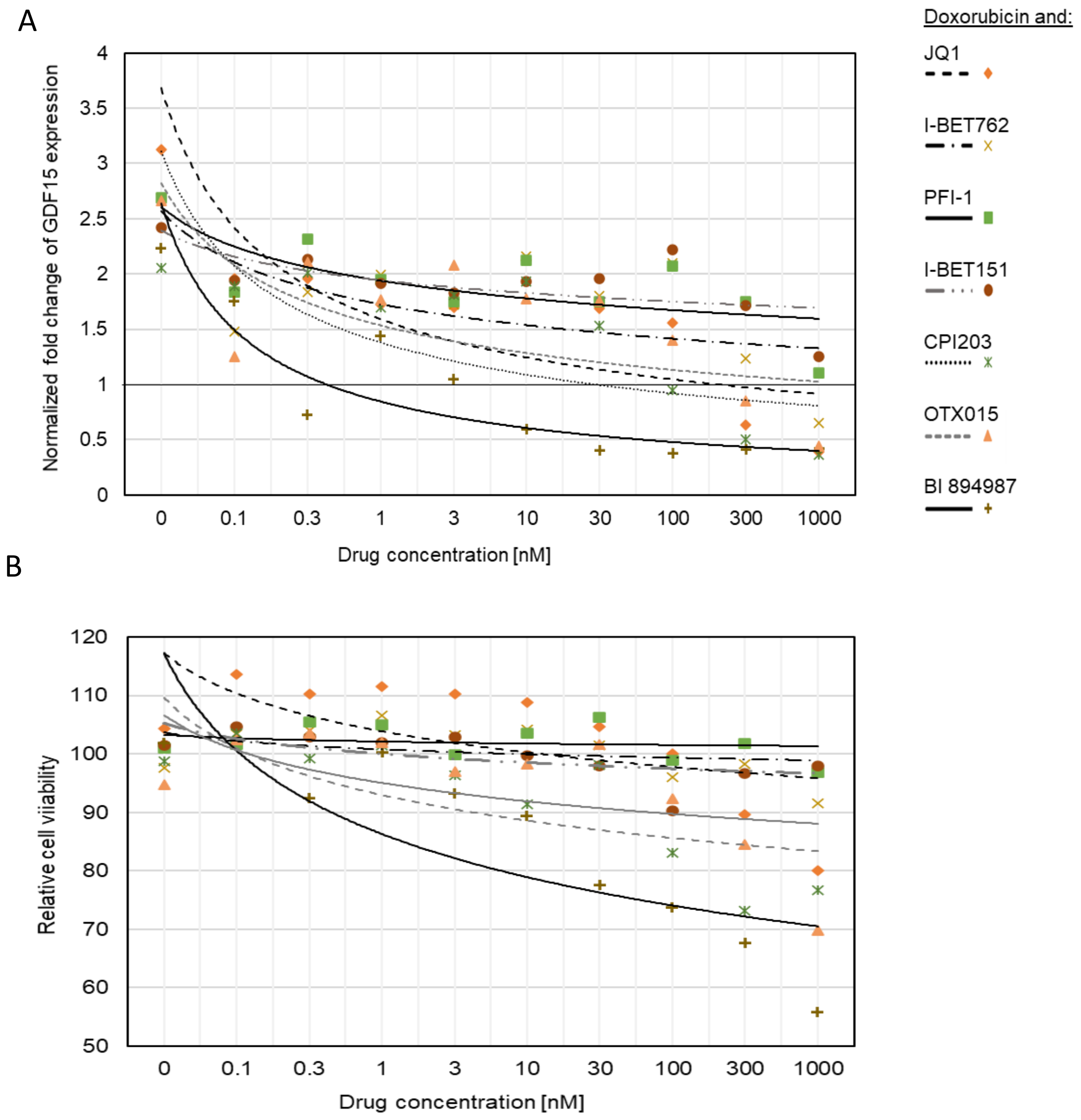

Supplementary Figure S6. (A) Fold-change of GDF15 secretion by hiPSC-CMs after simultaneous treatment with doxorubicin and seven BET Inhibitors for 48 h. Normalized to cell viability and non-treated control. Non-linear fit. (**B)** Effect of BET Inhibition on cell viability of doxorubicin treated hiPSC-CMs.

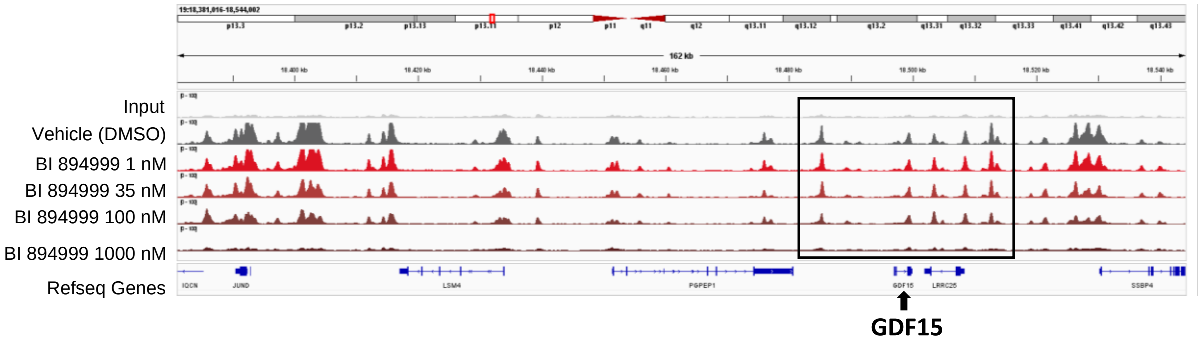

Supplementary Figure S7. Reduction of BRD4 bound to putative enhancers in close vicinity to the GDF15 gene locus by BI 894999.

Supplementary Tables

Supplementary Table S1. Characteristic properties of identified cell lines clusters of NCI-60, clustered based on GDF15 induction behavior upon treatment with 15 different anticancer drugs

		logFC	p-value
**Cluster A**			
Gene signatures	NIBR_TP53	8.52	3.30E-03
Hallmark gene sets	Notch	-6.84	5.00E-02
Gene expression	GDF15	0.14	4.25E-03
Correlation	GDF15 - BRD4	-0.55	3.21E-02
Protein expression	STAT3_pY705	-0.36	1.77E-02
	EGFR_pY1068	-0.61	2.83E-02
	p53	-0.66	3.52E-02
	Akt_pS473	-1.00	3.76E-02
**Cluster B**			
Protein expression	Chk2_pT68	-0.54	2.70E-04
	14-3-3_zeta	0.51	1.53E-03
	Lck	0.57	1.79E-02
	Bim(EP1036)	-0.66	3.18E-02
**Cluster C**			
Mutations	TP53	0.28	4.02E-03
Gene signatures	NIBR_TP53	-8.03	1.30E-03
Protein expression	c-Met	-0.28	9.20E-04
	BRCA2	-0.23	6.63E-03
	GSK3-alpha-beta	0.25	6.95E-03
	Cyclin_E1	0.48	1.07E-02
	p38_MAPK	0.40	3.60E-02
	p53	0.59	4.55E-02
**Cluster D**			
Mutations	BRAF	-0.79	5.86E-03
	PIK3CB	-0.71	9.41E-03
Hallmark gene sets	Myc Targets V2	-30.00	2.16E-02
Protein expression	PI3K-p85	-0.96	2.26E-05
	GSK3-alpha-beta	-0.48	1.01E-04
	eIF4G	1.55	1.08E-04
	VEGFR2	1.82	2.75E-04
	S6_pS235_S236	1.63	4.03E-04
	14-3-3_beta	-0.38	1.06E-03
	NF-kB-p65_pS536	1.40	1.12E-03
	eIF4E	-0.55	1.80E-03
	c-Met_pY1235	-0.21	2.93E-03
	EGFR	1.39	3.98E-03
	ER-alpha_pS118	-0.26	6.99E-03
	beta-Catenin	1.50	1.03E-02
	EGFR_pY1068	0.92	1.11E-02
	Notch1	0.45	1.11E-02
	p38_MAPK	-0.62	1.65E-02
	Akt	-0.59	3.27E-02
	FOXO3a_pS318_S321	-0.21	4.47E-02

Red: Higher in this cluster
Blue: Lower in this cluster

Supplementary Table S2. Molecular factors considered for correlation analysis

Mutations (in-house data)	Gene signatures	Gene sets [3]	Protein expression [4]	Gene expression (in-house data)
BRAF	NIBR IFN [5]	Wnt Beta Catenin	Akt	ARHGAP15
TIP3CB	NIBR P53 [6]	Unfold protein	AR	BDNF
PIK3CA	MERCK18 [7]	TNF alpha	ATM	BMP5
TP53	RAS84 [8]	PI3K	Bak	CALCR
MUC16	MPAS [9]	p53	Beta Catenin	CDK8
	TGREP2 [10]	Notch	BCL-2	CYP1B1
		MYC V1	Bim	DEPTOR
		Kras Up	BRCA2	ESR1
		Kras Dn	Caspase 7	FAM161A
		Estrogen Response Late	EGFR	FES
		Estrogen Response Early	ER alpha	FURIN
			FASN	GRB2
			G6PD	HIRA
			GATA3	HSPB3
			GSK3 alpha beta	IGFBP3
			Lck	IL16
			p14Arf	KRT19
			p27	LAD1
			p38_MAPK	LYL1
			p53	MAP3K3
			p70S6K	MAPT
			PI3K	MVB12B
			PRDX1	PCSK2
			PREX1	PREX1
			Rb	SCNN1B
			TTF1	SCNN1G
			VEGFR2	SLC9A3R1
				SLTRK4
				SULF1
				WISP2

Supplementary Table S3. Cancer cell lines used for BI 894999 treatment, cell line origin, authentication, cultivation, drug treatment, RNA isolation and sequencing

Cell line	Source	Date of acquisition	Date of STR authentication	BI 894999 treatment
Ty-82	JCRB1330	16.03.2021	14.06.2021	10 nM, 24h
10-15	As described in ref. [2]			
14169				
10326				
DLD-1	ATCC CCL-221 LOT 1658005	13.12.2002	13.07.2017	35 nM, 4 h
HCT-8	ATCC CCL-244 LOT 59313340	07.11.2014	12.01.2015	
HT55	ECACC 85061105 LOT 09K002	09.04.2014	16.12.2019	
LoVo	ATCC-CCL-229 LOT 59325448	11.04.2013	18.10.2018	
LS411N	ATCC CRL-2159 LOT 59948969	04.12.2013	29.07.2014	
LS513	ATCC CRL-2134 LOT 57761342	29.08.2011	21.02.2017	
NCI-H716	ATCC CCL-251 LOT 59057237	22.06.2011	12.04.2019	
SW620	ECACC 87051203 LOT 10G022	03.04.2013	12.08.2013	
SW948	ATCC CCL-237 LOT 59429140	18.04.2013	30.05.2013	
NCI-H69	ATCC HTB-119 LOT 3804337	17.06.2005	13.05.2015	100 nM, 4 h
NCI-H1048	ATCC CRL5853 LOT 61153909	31.10.2013	18.10.2018	
NCI-H2196	ATCC CRL-5932 LOT 57678465	19.05.2011	22.05.2013	
NCI-H2081	ATCC CRL-5920 LOT 57680855	27.02.2017	19.04.2017	
NCI-H510A	ATCC-HTB-184 LOT 63029159	06.12.2016	10.02.2018	
NCI-H889	ATCC CRL-5817 LOT 59887124	04.12.2013	12.12.2013	
DMS 454	ECACC 95062832	19.06.2013	23.09.2013	
DMS 53	ATCC CRL-2062 LOT 60687562	26.11.2013	10.07.2018	
COR-L95	ECACC 96020733 LOT 12B026	11.06.2013	07.05.2019	

Supplementary Table S4. Cell line groups for differential analysis of BET inhibitor responsiveness regarding GDF15 reduction upon treatment

Strong responsiveness<br>Reduction of GDF15 and activation of GDF11	Weak responsiveness<br>No or slight regulation of GDF15 and GDF11
10-15	COR-L95
10326	DLD-1
14169	DMS 454
HT55	DMS 53
LoVo	HCT-8
NCI-H2196	LS411N
NCI-H69	LS513
SW620	NCI-H1048
SW948	NCI-H716

Supplementary Table S5. Nodes of basic QIAGEN network upstream of GDF15 for pathway analysis

Symbol	Entrez Gene Name
ABL1	ABL proto-oncogene 1, non-receptor tyrosine kinase
Akt	
AMPK	
BAX	BCL2 associated X, apoptosis regulator
BCL2	BCL2 apoptosis regulator
BCL2L11	BCL2 like 11
Betacatenin/TCF	
BRAF	B-Raf proto-oncogene, serine/threonine kinase
BRCA2	BRCA2 DNA repair associated
CCND1	cyclin D1
CCNE1	cyclin E1
CDK8	cyclin dependent kinase 8
CTNNB1	catenin beta 1
CYP1B1	cytochrome P450 family 1 subfamily B member 1
EGFR	epidermal growth factor receptor
EGR1	early growth response 1
EIF4EBP1	eukaryotic translation initiation factor 4E binding protein 1
ESR1	estrogen receptor 1
FOS	Fos proto-oncogene, AP-1 transcription factor subunit
FOXO3	forkhead box O3
GDF11	growth differentiation factor 11
GDF15	growth differentiation factor 15
GSK3B	glycogen synthase kinase 3 beta
Jnk	
KDR	kinase insert domain receptor
LCK	LCK proto-oncogene, Src family tyrosine kinase
Mapk	
MAPT	microtubule associated protein tau
MDM2	MDM2 proto-oncogene
MET	MET proto-oncogene, receptor tyrosine kinase
MTOR	mechanistic target of rapamycin kinase
MYC	MYC proto-oncogene, bHLH transcription factor
NFkB (complex)	
NOTCH1	notch receptor 1
NOTCH2	notch receptor 2
NR5A2	nuclear receptor subfamily 5 group A member 2
P38 MAPK	
PI3K (complex)	
RAS	
SMAD2	SMAD family member 2
SMAD3	SMAD family member 3
STAT3	signal transducer and activator of transcription 3
TNFRSF10A	TNF receptor superfamily member 10a
TP53	tumor protein p53

Supplementary Materials and Methods

Data collection of cancer cell line properties

Gene expression data of NCI-60 upon treatment with 15 anticancer compounds were collected from NCI-60 Transcriptional Pharmacodynamics Workbench [11]. We collected basal protein expression values for all studied cancer cell lines from reported Reverse Phase Protein Array data [4]. Drug sensitivity data were taken from Drug Repurposing Hub and indicated in activity areas (1 – integral of the dose response curve) [1,12]. Gene dependency data were extracted from CRISPR and shRNA gene knock-out/down studies using CERES or RSA algorithms [13–16]. Furthermore, we used cell line specific data of a cancer metabolome study that quantified metabolites using liquid chromatography mass spectrometry [17]. IC50 values of tested BET inhibitors were taken from ref. [18–23]. We additionally calculated six reported gene signatures and 50 hallmark gene set scores to deduce specific protein activities and pathway activities from gene and protein expression data [3,5–10].

Supplementary References

1. Corsello, S. M. *et al.* The Drug Repurposing Hub: a next-generation drug library and information resource. *Nat Med* 23, 405–408 (2017).

2. Tontsch-Grunt, U. *et al.* Therapeutic impact of BET inhibitor BI 894999 treatment: backtranslation from the clinic. *Brit J Cancer* 127, 577–586 (2022).

3. Liberzon, A. *et al.* The Molecular Signatures Database Hallmark Gene Set Collection. *Cell Syst* 1, 417–425 (2015).

4. Ghandi, M. *et al.* Next-generation characterization of the Cancer Cell Line Encyclopedia. *Nature* 569, 503–508 (2019).

5. Liu, H. *et al.* Tumor-derived IFN triggers chronic pathway agonism and sensitivity to ADAR loss. *Nat Med* 25, 95–102 (2019).

6. Jeay, S. *et al.* A distinct p53 target gene set predicts for response to the selective p53–HDM2 inhibitor NVP-CGM097. *Elife* 4, e06498 (2015).

7. Ayers, M. *et al.* IFN-γ–related mRNA profile predicts clinical response to PD-1 blockade. *J Clin Invest* 127, 2930–2940 (2017).

8. East, P. *et al.* Oncogenic RAS activity predicts response to chemotherapy and outcome in lung adenocarcinoma. *Biorxiv* 2021.04.02.437896 (2021) doi:10.1101/2021.04.02.437896.

9. Wagle, M.-C. *et al.* A transcriptional MAPK Pathway Activity Score (MPAS) is a clinically relevant biomarker in multiple cancer types. *Npj Precis Oncol* 2, 7 (2018).

10. Reddy, A. *et al.* Gene Expression Ratios Lead to Accurate and Translatable Predictors of DR5 Agonism across Multiple Tumor Lineages. *Plos One* 10, e0138486 (2015).

11. Monks, A. *et al.* The NCI Transcriptional Pharmacodynamics Workbench: a tool to examine dynamic expression profiling of therapeutic response in the NCI-60 cell line panel. *Cancer Res* 78, canres.0989.2018 (2018).

12. Barretina, J. *et al.* The Cancer Cell Line Encyclopedia enables predictive modeling of anticancer drug sensitivity. *Nature* 483, 603–607 (2012).

13. Meyers, R. M. *et al.* Computational correction of copy number effect improves specificity of CRISPR–Cas9 essentiality screens in cancer cells. *Nat Genet* 49, 1779–1784 (2017).

14. McDonald, E. R. *et al.* Project DRIVE: A Compendium of Cancer Dependencies and Synthetic Lethal Relationships Uncovered by Large-Scale, Deep RNAi Screening. *Cell* 170, 577-592.e10 (2017).

15. Behan, F. M. *et al.* Prioritization of cancer therapeutic targets using CRISPR–Cas9 screens. *Nature* 568, 511–516 (2019).

16. Dempster, J. M. *et al.* Extracting Biological Insights from the Project Achilles Genome-Scale CRISPR Screens in Cancer Cell Lines. *Biorxiv* 720243 (2019) doi:10.1101/720243.

17. Li, H. *et al.* The landscape of cancer cell line metabolism. *Nat Med* 25, 850–860 (2019).

18. Ran, X. *et al.* Structure-Based Design of γ-Carboline Analogues as Potent and Specific BET Bromodomain Inhibitors. *J Med Chem* 58, 4927–4939 (2015).

19. Shadrick, W. R. *et al.* Exploiting a water network to achieve enthalpy-driven, bromodomain-selective BET inhibitors. *Bioorganic & Medicinal Chemistry* 26, 25–36 (2018).

20. Ali, I., Lee, J., Go, A., Choi, G. & Lee, K. Discovery of novel [1,2,4]triazolo[4,3-a]quinoxaline aminophenyl derivatives as BET inhibitors for cancer treatment. *Bioorganic & Medicinal Chemistry Letters* 27, 4606–4613 (2017).

21. Zhou, B. *et al.* Discovery of a Small-Molecule Degrader of Bromodomain and Extra-Terminal (BET) Proteins with Picomolar Cellular Potencies and Capable of Achieving Tumor Regression. *J Med Chem* 61, 462–481 (2018).

22. Devaiah, B. N. *et al.* BRD4 is an atypical kinase that phosphorylates serine2 of the RNA polymerase II carboxy-terminal domain. *Proc National Acad Sci* 109, 6927–6932 (2012).

23. Zhao, L. *et al.* Fragment-Based Drug Discovery of 2-Thiazolidinones as BRD4 Inhibitors: 2. Structure-Based Optimization. *J Med Chem* 58, 1281–1297 (2015).
